# Matrine suppresses KRAS‐driven pancreatic cancer growth by inhibiting autophagy‐mediated energy metabolism

**DOI:** 10.1002/1878-0261.12324

**Published:** 2018-06-11

**Authors:** Young‐ra Cho, Ji Hyeon Lee, Ji Hye Kim, So‐Yeon Lee, Suna Yoo, Min‐kyo Jung, Su Jung Kim, Hyun Ju Yoo, Chang‐Gi Pack, Jin Kyung Rho, Jaekyoung Son

**Affiliations:** ^1^ Department of Biomedical Sciences Asan Medical Center AMIST University of Ulsan College of Medicine Seoul South Korea; ^2^ Asan Institute for Life Sciences Asan Medical Center University of Ulsan College of Medicine Seoul South Korea; ^3^ Department of Convergence Medicine Asan Institute of Life Sciences Asan Medical Center University of Ulsan College of Medicine Seoul South Korea; ^4^ Department of Convergence Medicine Asan Medical Center University of Ulsan College of Medicine Seoul South Korea; ^5^ Department of Convergence Medicine Asan Medical Center AMIST University of Ulsan College of Medicine Seoul South Korea

**Keywords:** autophagy, KRAS, lysosomal protease, metabolism, pancreatic cancer

## Abstract

Matrine is a natural compound extracted from the herb *Sophora flavescens* Ait which is widely used in traditional Chinese medicine for treating various diseases. Recently, matrine was reported to have antitumor effects against a variety of cancers without any obvious side effects; however, the molecular mechanisms of its antiproliferative effects on cancer are unclear. Here, we report that matrine inhibits autophagy‐mediated energy metabolism, which is necessary for pancreatic cancer growth. We found that matrine significantly reduces pancreatic cancer growth *in vitro* and *in vivo* by insufficiently maintaining mitochondrial metabolic function and energy level. We also found that either pyruvate or α‐ketoglutarate supplementation markedly rescues pancreatic cancer cell growth following matrine treatment. Inhibition of mitochondrial energy production results from matrine‐mediated autophagy inhibition by impairing the function of lysosomal protease. Matrine‐mediated autophagy inhibition requires stat3 downregulation. Furthermore, we found that the antitumor effect of matrine on pancreatic cancer growth depends on the mutation of the KRAS oncogene. Together, our data suggest that matrine can suppress the growth of KRAS‐mutant pancreatic cancer by inhibiting autophagy‐mediated energy metabolism.

**Abbreviations**
CQ: chloroquine
HPDE: human pancreatic ductal cells
LC‐MS/MS: liquid chromatography–tandem mass spectrometry
PDAC: pancreatic ductal adenocarcinoma

## 1 Introduction

Pancreatic cancer is highly lethal with a poor prognosis. Pancreatic ductal adenocarcinoma (PDAC) is the most common malignant pancreatic tumor and is extremely aggressive with a 5‐year survival rate of only 8.0%.(Siegel *et al*., 2016) The most effective treatment for PDAC is surgical resection; however, because pancreatic cancer is usually not detected before it has already spread beyond the pancreas, > 80% of patients cannot be cured with surgery. (Hidalgo, 2010) Cytotoxic chemotherapies, targeted agents, and radiotherapy are other options for treating PDAC; however, PDAC is highly resistant to these treatments, which explains why these options are largely ineffective and why PDAC has the lowest survival rate of any major type of cancer.(Ben‐Josef and Lawrence, 2008) Thus, there is an urgent need for new therapeutic targets to treat this devastating disease.

There has been a resurgence of interest over the past decade in understanding the altered cellular metabolic pathways in cancer cells and how such dependencies can be targeted for therapeutic gain. Alterations in the cellular metabolic pathways in cancer are considered a hallmark of the disease.(Hanahan and Weinberg, 2011) The metabolic requirements and pathways of cancer cells are considerably different from those of normal cells because proliferating cancer cells must contain increased amounts of basic cellular building blocks, such as carbohydrates and amino acids, as well as increases in nucleotides and lipid mass to support their high rates of proliferation.(Vander Heiden *et al*., 2009) During the process of tumorigenesis, genetic and epigenetic alterations fine‐tune cancer cell metabolism to optimize growth and survival in the tumor microenvironment.(Ward and Thompson, 2012) Thus, the considerable metabolic differences between cancer cells and normal cells is a potential target of cancer therapy(Vander Heiden, 2011); however, because normal cells might use the same metabolic pathways as cancer cells, a successful therapeutic index remains a major challenge to the development of effective cancer therapies targeting their specific metabolic pathways. Therefore, a better understanding of the adaptive metabolic changes of cancer cells is required for success in targeting cancer metabolism.

Autophagy, an evolutionarily conserved mechanism, is a critical cellular pathway to maintain cellular homeostasis by degrading unnecessary proteins and organelles.(Mizushima *et al*., 2008) This degradation results in the release of nucleosides, amino acids, proteins, lipids, fatty acids, and sugars into the cell's cytoplasm for recycling.(White *et al*., 2015) Emerging evidence has exhibited that some cancers have high basal levels of autophagy or induce autophagy for survival, suggesting that autophagy plays a role as a tumor promoter.(Guo *et al*., 2013; Yang *et al*., 2011, 2014) One of the mechanisms by which autophagy promotes cancer is by maintaining the metabolic functions of mitochondria and energy homeostasis to meet the metabolic demands of increased cell growth and proliferation.(Galluzzi *et al*., 2014) Thus, cancer cells rely more heavily on autophagy‐mediated metabolism than do normal cells, indicating that autophagy inhibition can be a therapeutic target for cancer therapy.

Matrine is an alkaloid compound that is found in the herb root *Sophora flavescens* Ait, a medicinal herb used in traditional Chinese medicine.(Luo *et al*., 2007) It has been reported that matrine has pharmacological properties against various diseases, such as anti‐inflammatory, antiallergic, antivirus, antifibrotic, and cardiovascular protective effects.(Li *et al*., 2009; Liu *et al*., 2007; Long *et al*., 2004; Zhang *et al*., 2001) Furthermore, recent evidence has exhibited that matrine exerts antitumor effects on various tumor cells, including cervical cancer, leukemia, pancreatic cancer, gastric cancer, lung cancer, and breast cancer(Dai *et al*., 2009; Liu *et al*., 2010; Yu *et al*., 2009; Zhang *et al*., 2007, 2009, 2012); however, the precise mechanisms underlying this antitumor activity remain largely unknown.

In this study, we demonstrated for the first time inhibitory effect of matrine on cancer metabolism. We found that matrine inhibits autophagy by impairing the function of lysosomal protease, leading to a significant reduction in pancreatic cancer growth and proliferation by suppressing autophagy‐mediated mitochondrial metabolic demands. Furthermore, we revealed that stat3 is essential for matrine‐mediated autophagy inhibition and that matrine suppresses the growth of KRAS‐mutant PDAC, but not KRAS‐wt PDAC. Thus, our data suggest that matrine might be an effective candidate as a therapeutic agent for KRAS‐mutant pancreatic cancer.

## 2 Results

### 2.1 Matrine inhibits pancreatic cancer growth

**Figure 1:**
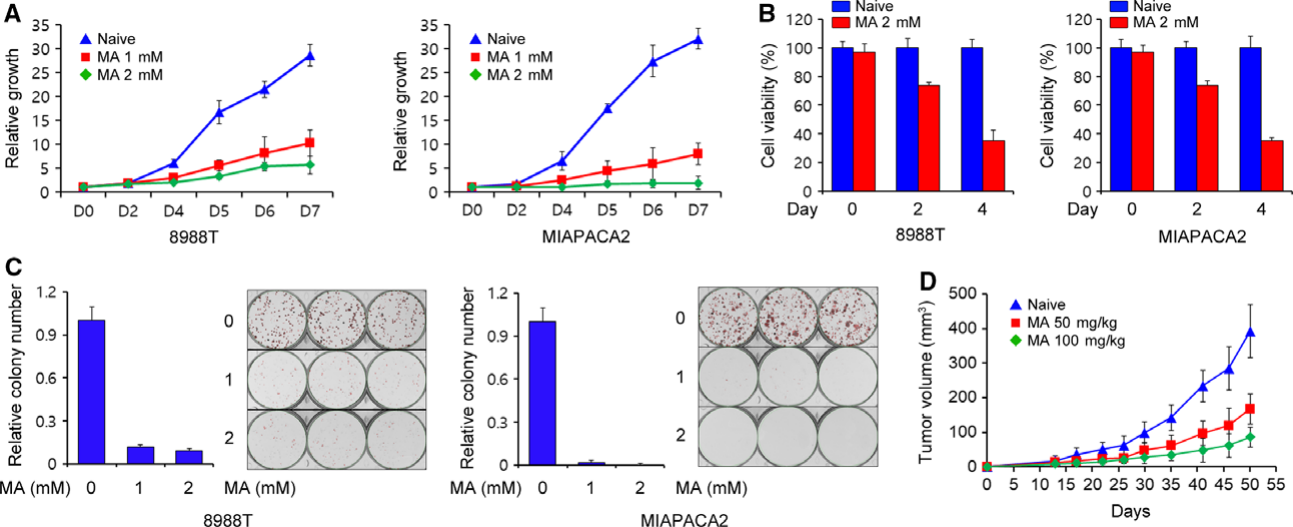
Effect of matrine on pancreatic ductal adenocarcinoma (PDAC) growth. (A) PDAC cells were treated with the indicated concentration of matrine and assayed for cell growth. (B) PDAC cells were treated with 2‐mm matrine for 24 h and assayed for cell viability. (C) Colony‐forming assays were performed to assess the ability of matrine to inhibit PDAC growth. Error bars represent the SD of triplicate wells from a representative experiment. (D) Subcutaneous 8988T‐driven tumors were established in severe combined immunodeficiency (SCID) mice. Matrine (50 mg·kg^−1^ or 100 mg·kg^−1^) was administered daily by intraperitoneal injection. Data are shown as the mean of five mice in each group ± SEM.

To investigate the effect of matrine on PDAC growth, PDAC cells were first treated with 1‐ or 2‐mm matrine. As shown in Fig. 1A, matrine treatment significantly decreased PDAC growth in a dose‐dependent manner. To further confirm that matrine reduced PDAC growth, we treated PDAC cells with matrine and assayed for cell viability. Consistent with the growth curve, matrine treatment resulted in a profound reduction in the proliferation of PDAC cells (Fig. 1B). In addition, PDAC cell colony formation was markedly inhibited in the presence of matrine (Fig. 1C). We then tested the impact of matrine on the growth and proliferation of nontransformed human pancreatic ductal cells (HPDE). Matrine significantly affected the growth and proliferation of HPDE (Fig. S1A,B), suggesting that the antitumor effect of matrine is not tumor‐specific in PDAC. As further confirmation of the antitumor effect of matrine on PDAC growth, we assessed its ability to grow *in vivo* as a xenograft. As shown in Fig. 1D, matrine treatment robustly diminished tumor growth in a dose‐dependent manner; therefore, *in vitro* and *in vivo* data indicate that matrine exerts antitumor activities on pancreatic cancer cells.

### 2.2 Matrine suppresses PDAC growth by depriving tricarboxylic acid cycle substrates

**Figure 2:**
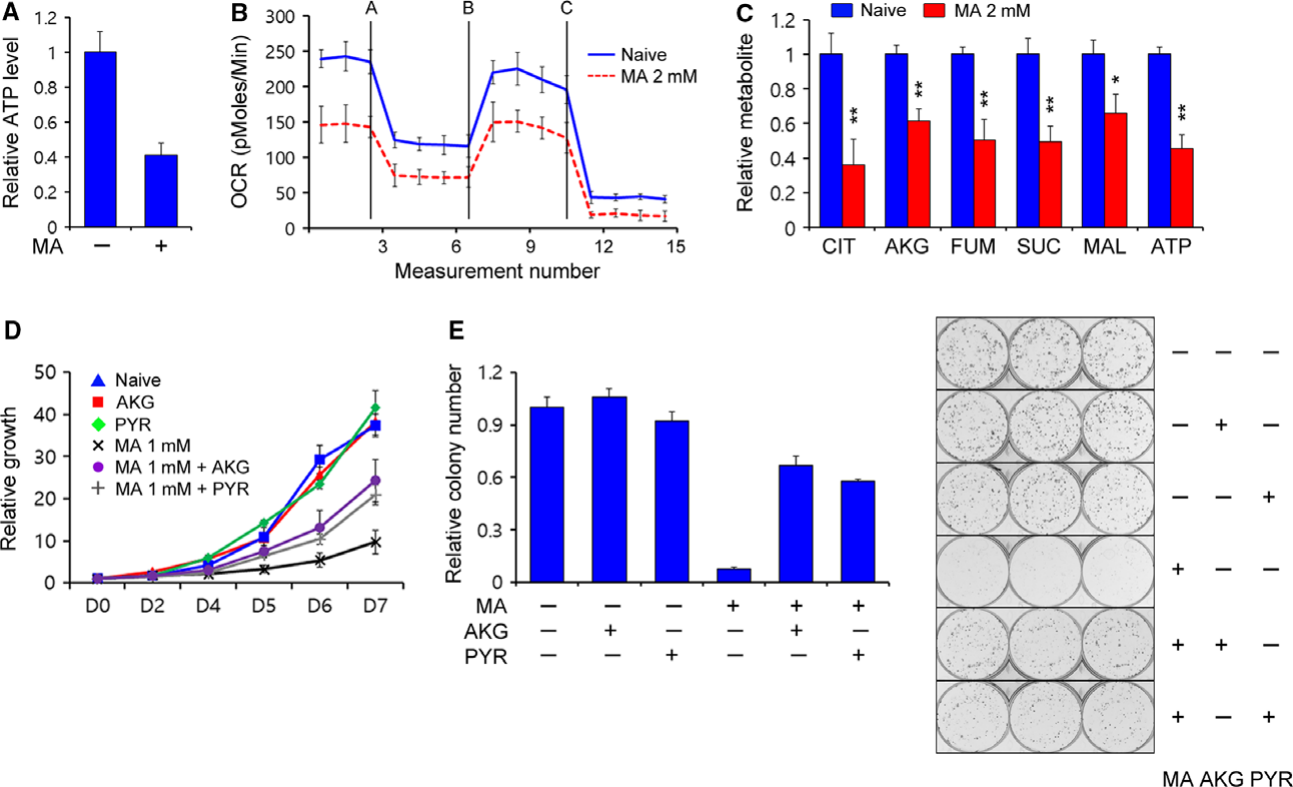
Matrine reduces levels of tricarboxylic acid (TCA) cycle intermediates. (A) 8988T cells were treated with 2‐mm matrine for 24 h and assayed for intracellular adenosine triphosphate (ATP). (B) Oxygen consumption rates were measured with an extracellular flux analyzer. 8988T cells were treated with 2‐mm matrine. Cells were sequentially treated with 2‐μm oligomycin, 5‐μm FCCP, and 2‐μm rotenone. (C) TCA metabolite pools in 8988T cells treated with 2‐mm matrine were analyzed using LC‐MS/MS. Error bars represent the SD of three independently prepared samples. (D and E) 8988T cells were treated with 1‐mm matrine in the presence of either 7‐mm methyl pyruvate or 7‐mm dimethyl α‐ketoglutarate and assayed for cell growth (D) and assayed for colony formation (E). Error bars represent the SD of triplicate wells from a representative experiment. CIT, citrate; AKG, alpha‐ketoglutarate; FUM, fumalate; SUC, succinate; MAL, malate; ATP, adenosine triphosphate. *, *P *< 0.05; **, *P *< 0.01.

Compared with normal differentiated cells, proliferating cancer cells need several nutrients, such as nucleotides, amino acids, and lipids, in large amounts to meet the increased metabolic demands of proliferating cells.(Lyssiotis *et al*., 2013) As such, cancer cells are also much more susceptible than normal cells to an insufficient supply of cellular building blocks by the destruction of metabolic pathways. Thus, we first examined the energy levels in the absence or presence of matrine. As shown in Fig. 2A, adenosine triphosphate (ATP) levels were significantly reduced in the presence of matrine. Oxygen consumption also decreased markedly after matrine treatment (Fig. 2B). We next investigated the effect of matrine on mitochondrial metabolism using targeted liquid chromatography–tandem mass spectrometry (LC‐MS/MS) metabolomic analysis to explore the direct effect of matrine on mitochondrial ATP production. We found that matrine treatment led to a significant reduction in the levels of tricarboxylic acid (TCA) cycle intermediates (Fig. 2C). To test whether matrine‐mediated reduction in PDAC growth is a result of the insufficient maintenance of mitochondrial ATP production, we attempted to reverse this reduction by supplementing the cells with either pyruvate or α‐ketoglutarate. As shown in Fig. 2D,E, the addition of either pyruvate or α‐ketoglutarate markedly rescued PDAC cell growth. We next tested the effect of matrine on mitochondrial metabolism of HPDE. In contrast to PDAC, matrine had no significant impact on the ATP levels of HPDE (Fig. S1C), and the addition of α‐ketoglutarate did not rescue the HPDE cell growth (Fig. S1D). Together, these data suggest that matrine inhibits PDAC growth by suppressing mitochondrial metabolism.

### 2.3 Matrine accumulates autophagic vacuoles

**Figure 3:**
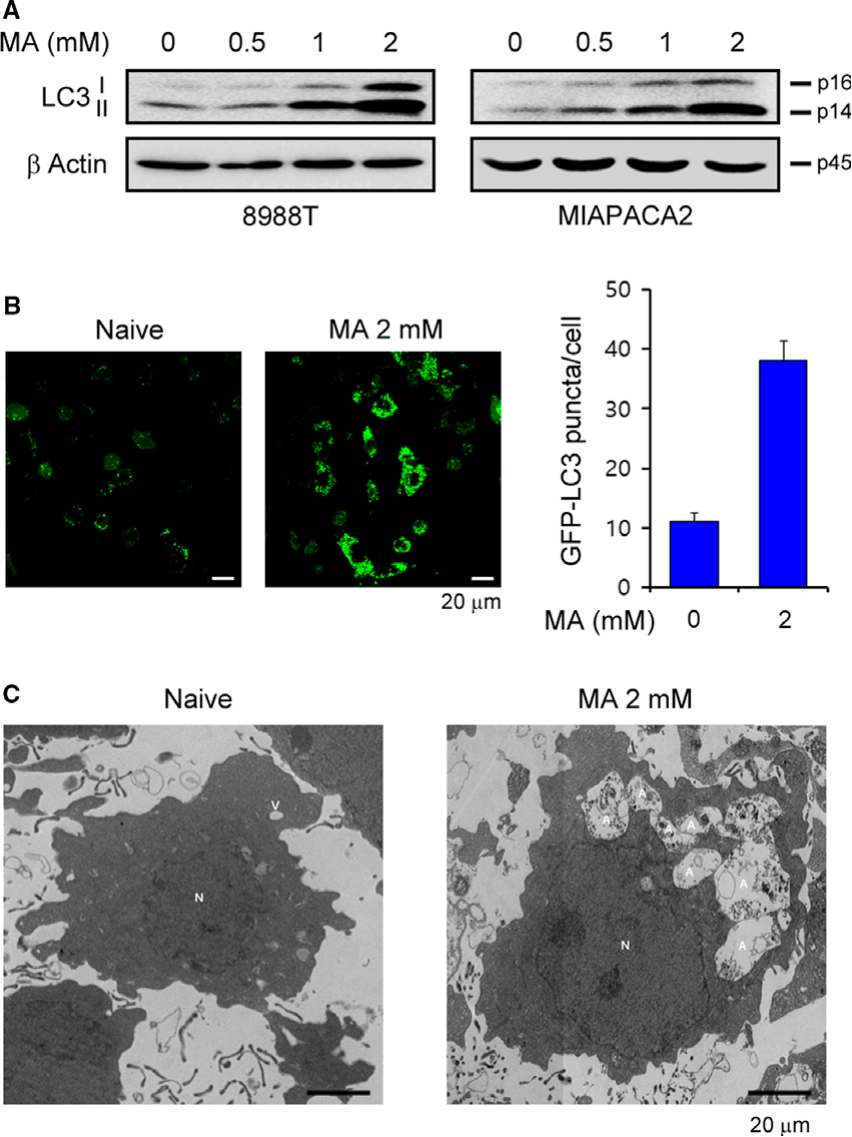
Effect of matrine on autophagy. (A) PDAC cells were treated with the indicated concentration of matrine for 24 h and immunoblotted with the indicated antibodies. (B) 8988T cells expressing GFP‐LC3 were treated with 2‐mm matrine for 24 h and analyzed for LC3 dots. (C) Autophagic vacuoles in 8988T cells treated with 2‐mm matrine for 24 h were analyzed by transmission electron microscopy. Notes: N, nucleus; L, lipid; A, autophagic vacuoles.

We next investigated the mechanisms by which matrine suppresses mitochondrial metabolism. Several studies have reported that autophagy plays a critical role in cancer metabolism.(Galluzzi *et al*., 2014; Goldsmith *et al*., 2014; White *et al*., 2015) In addition, studies have shown that autophagy is essential for maintaining intracellular glutamine levels, which is a primary carbon source for the TCA cycle in pancreatic cancer.(Seo *et al*., 2016; Ying *et al*., 2012) Thus, we tested whether matrine has an effect on the mechanisms that drive autophagy. Correspondingly, matrine treatment resulted in a significant increase in LC3‐II levels (Fig. 3A) and robustly increased the number of GFP‐LC3 puncta compared with that in cells cultured under normal conditions (Fig. 3B). In addition, the number of autophagic vacuoles increased after matrine treatment (Fig. 3C). In contrast to PDAC, there was no alteration in LC3‐II levels upon matrine treatment (Fig. S1E), indicating that matrine suppresses the HPDE growth by a different mechanism. Together, these data indicate that matrine treatment leads to the accumulation of autophagic vacuoles in PDAC cells.

### 2.4 Matrine inhibits autophagic degradation by impairing the function of lysosomal proteases

**Figure 4:**
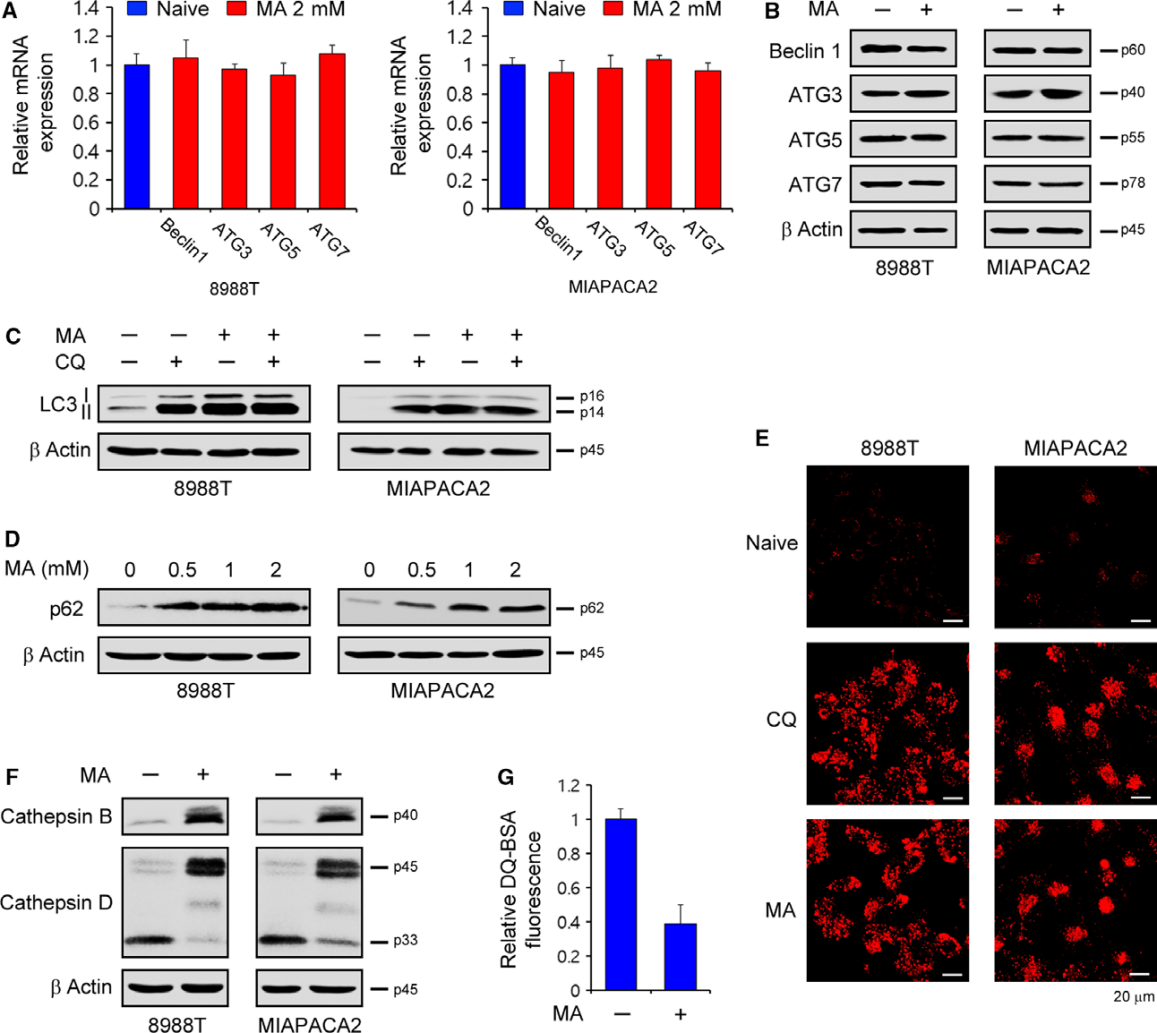
Matrine inhibits the maturation of cathepsins. (A and B) Expression levels of Beclin1, ATG3, ATG5, and ATG7 were determined by quantitative reverse transcription polymerase chain reaction (qRT‐PCR) and western blotting in PDAC cells treated with 2‐mm matrine for 24 h. Error bars represent the SD of triplicate wells from a representative experiment. (C) PDAC cells were treated with 2‐mm matrine for 24 h in the absence or presence of 10‐μm chloroquine (CQ) and immunoblotted with the indicated antibodies. (D) PDAC cells were treated with the indicated concentration of matrine for 24 h and immunoblotted with p62. (E) LysoTracker staining was performed on PDAC cells treated with either 10‐μm CQ or 2‐mm matrine for 24 h. (F) PDAC cells were treated with 2‐mm matrine for 24 h and immunoblotted with the indicated antibodies. (G) 8988T cells were treated with 2‐mm matrine for 24 h and assayed for DQ‐BSA cleavage activity.

The accumulation of autophagic vacuoles can result from either an increase in their formation by inducing autophagic flux or a decrease in their rate of degradation by inhibiting the formation of autolysosomes.(Amaravadi *et al*., 2016) To assess the effect of matrine on autophagic flux, we first measured mRNA and the protein levels of autophagy core genes after matrine treatment. As shown in Fig. 4A,B, matrine treatment had no significant effect on the transcriptional and translational levels of autophagy core genes, indicating that matrine treatment might impair autophagosome degradation, which is the later stage in the autophagic process. To address this issue, PDAC cells were treated with chloroquine (CQ), an inhibitor of lysosomal acidification, in the absence or presence of matrine. CQ's inhibition of autophagosome degradation did not further accumulated LC3‐II levels under matrine treatment (Fig. 4C), but the levels of p62 markedly increased in response to matrine treatment in a dose‐dependent manner (Fig. 4D). Moreover, consistent with the result of CQ treatment, matrine treatment resulted in an increase in LysoTracker signal (Fig. 4E), indicating an aggregation of autophagosomes.

Given that the inhibition of lysosomal proteases has been causally linked to the impairment of autophagosome degradation (Fortunato and Kroemer, 2009; Kaminskyy and Zhivotovsky, 2012) and the inhibition of cathepsins, well‐known lysosomal proteases, and has been shown to disrupt autophagosome degradation, (Jung *et al*., 2015; Wang *et al*., 2013) we speculated that matrine treatment might inhibit the activation of cathepsins. Interestingly, pro‐forms of cathepsins B and D, two main cathepsins, dramatically increased after matrine treatment (Fig. 4F). Consistent with this result, matrine treatment significantly inhibits the activity of lysosomal protease (Fig. 4G). Together, these data suggest that matrine disrupts the function of lysosomal protease by inhibiting the formation of mature forms of cathepsins.

### 2.5 Stat3 is required for matrine‐mediated autophagy inhibition

**Figure 5:**
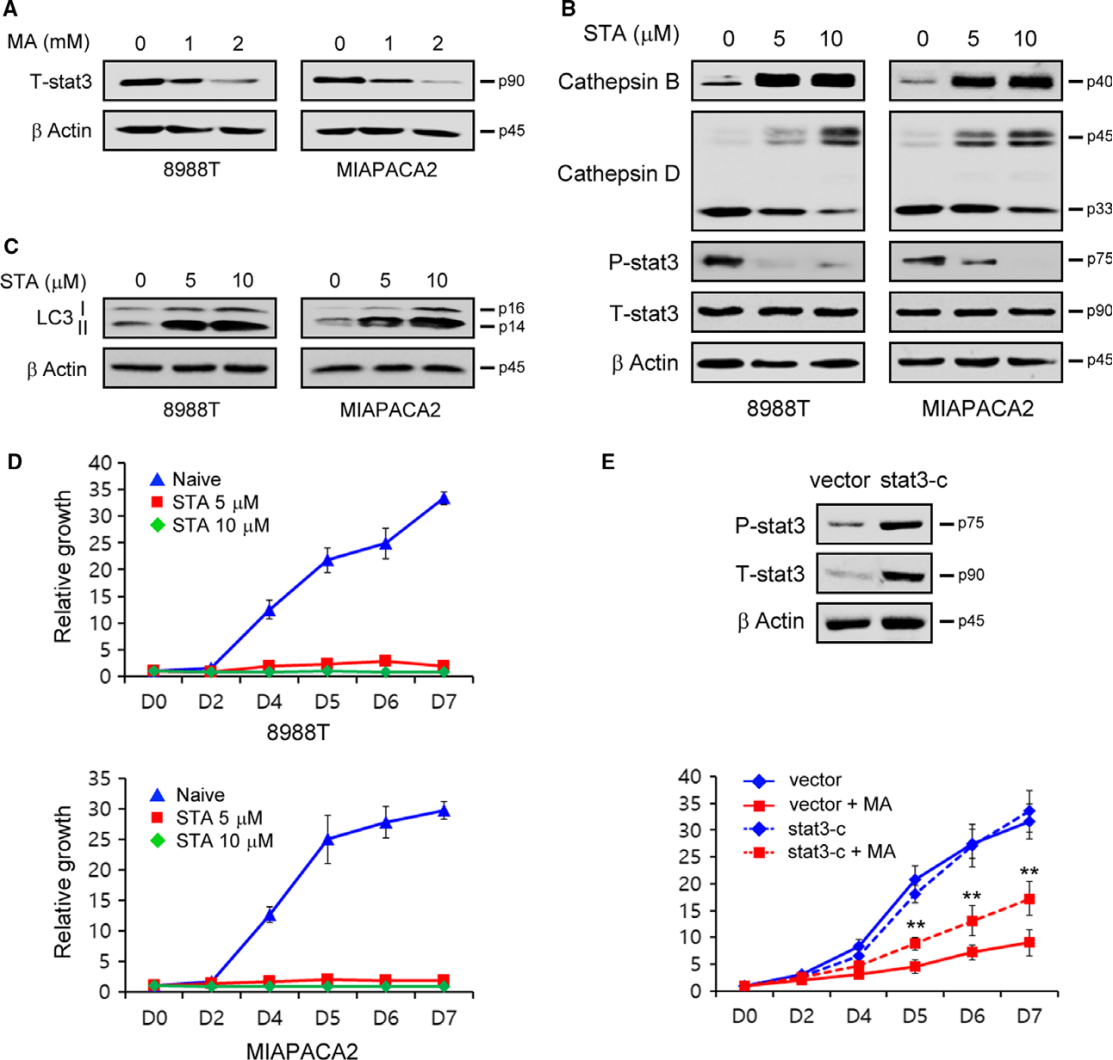
Matrine inhibits the maturation of cathepsins by reducing Stat3. (A) PDAC cells were treated with the indicated concentration of matrine for 24 h and immunoblotted with Stat3. (B and C) PDAC cells were treated with the indicated concentration of stattic for 24 h and immunoblotted with the indicated antibodies. (D) PDAC cells were treated with the indicated concentration of stattic and assayed for cell growth. (E) 8988T cells expressing a vector or constitutively active form of Stat3 (Stat3‐c) were treated with 1‐mm matrine and assayed for cell growth. Western blotting confirmed the overexpression of Stat3‐c. Error bars represent the SD of triplicate wells from a representative experiment. *P *< 0.05;**

We next explored the mechanism by which matrine inhibits the formation of mature forms of cathepsins and found that matrine downregulates the expression levels of Stat3 (Fig. 5A). We next examined whether matrine‐mediated Stat3 downregulation inhibits the formation of mature forms of cathepsins. Consistent with the matrine treatment data, treatment with the Stat3 inhibitor, stattic, significantly disrupted the formation of mature forms of cathepsins B and D (Fig. 5B). Stattic treatment also resulted in an increase in LC3‐II levels (Fig. 5C) and significantly decreased PDAC growth in a dose‐dependent manner (Fig. 5D). To further confirm that Stat3 downregulation is required for matrine‐mediated cell growth reduction, we attempted to rescue that reduction by overexpressing Stat3. As shown in Fig. 5E, overexpression of Stat3 significantly rescued PDAC growth. Thus, these data demonstrate that downregulating Stat3 can impair PDAC growth by inhibiting the activation of cathepsins.

### 2.6 The inhibitory effect of matrine on PDAC growth depends on KRAS mutation

**Figure 6:**
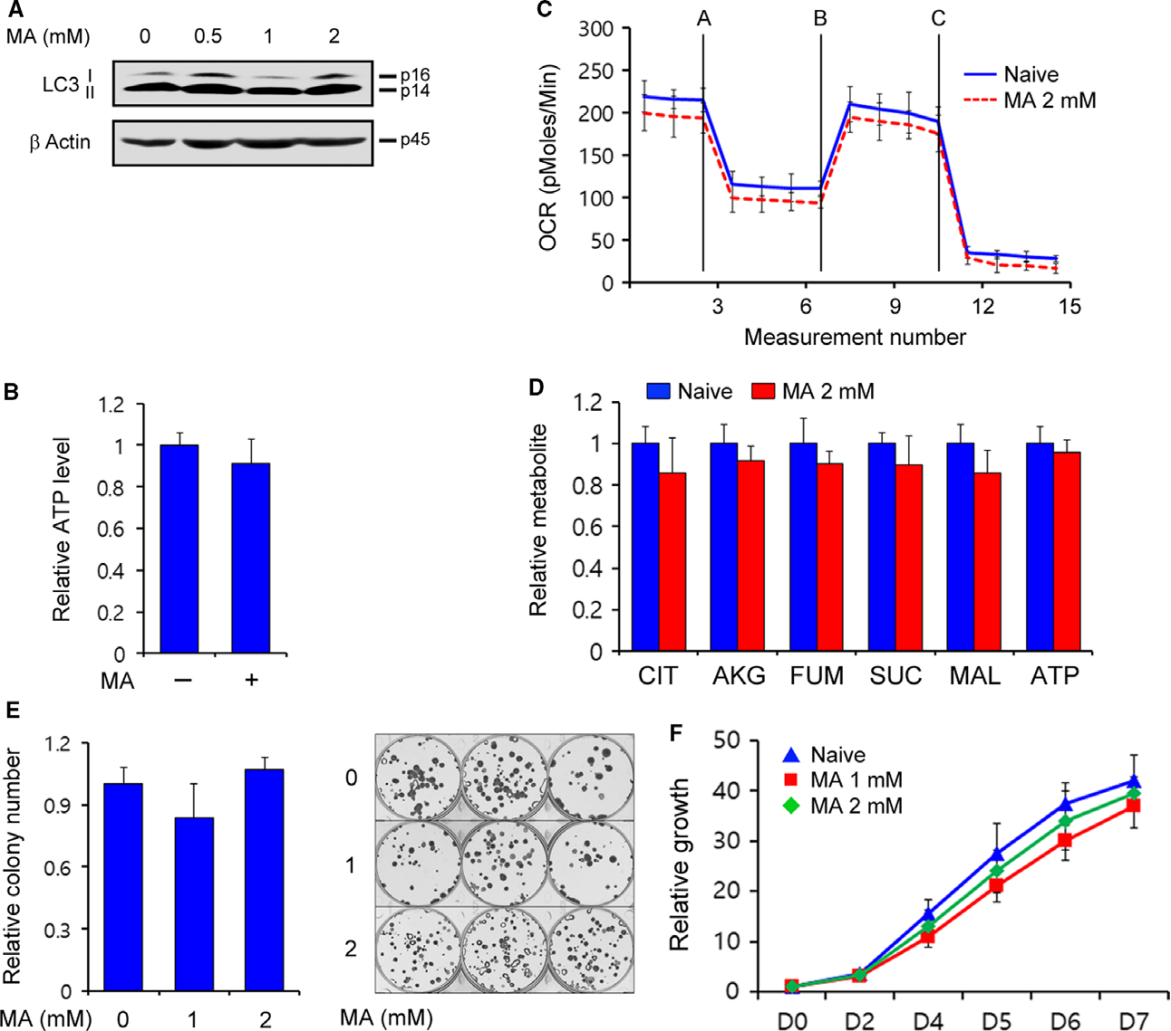
Matrine does not inhibit growth of KRAS‐wt PDAC. (A) BXPC3 cells were treated with the indicated concentration of matrine for 24 h and immunoblotted with LC3. (B) BXPC3 cells were treated with 2‐mm matrine for 24 h and assayed for intracellular ATP. (C) Oxygen consumption rates were measured using an extracellular flux analyzer. BXPC3 cells were treated with 2‐mm matrine. Cells were sequentially treated with 2‐μm oligomycin, 5‐μm FCCP, and 2‐μm rotenone. (D) TCA metabolite pools in BXPC3 cells treated with 2‐mm matrine were analyzed LC‐MS/MS. Error bars represent the SD of three independently prepared samples. (E) Colony‐forming assays were performed to assess the ability of matrine to inhibit BXPC3 growth. (F) BXPC3 cells were treated with the indicated concentration of matrine and assayed for cell growth. Error bars represent the SD. of triplicate wells from a representative experiment.

Considering the previous studies that showed that the oncogene KRAS plays a critical role in reprogramming PDAC metabolism (Son *et al*., 2013; Ying *et al*., 2012) and that KRAS‐driven autophagy also plays a key role in multiple aspects of PDAC metabolism, (Bryant *et al*., 2014; Guo *et al*., 2011; Yang *et al*., 2011) we speculated that the matrine‐mediated reduction of PDAC growth is dependent on KRAS mutation. We observed that matrine treatment had no effect on LC3‐II levels in BXPC3 cells, which harbor a KRAS‐wt (Fig. 6A). In contrast to the matrine results of KRAS‐mutant PDAC cells, there was no significant change in ATP levels (Fig. 6B) and oxygen consumption rate (Fig. 6C) in BXPC3 cells after matrine treatment. Consistent with these data, the levels of TCA cycle intermediates were not significantly altered in BXPC3 after matrine treatment (Fig. 6D). Furthermore, matrine treatment had no significant effect on colony formation (Fig. 6E) and growth of KRAS‐wt PDAC (Fig. 6F). Together, these data suggest that KRAS mutation is required for matrine‐mediated PDAC growth inhibition.

## 3 Discussion

Matrine has been shown to exert antitumor effects on a variety of cancers, including pancreatic cancer (Dai *et al*., 2009; Liu *et al*., 2010; Yu *et al*., 2009; Zhang *et al*., 2007, 2009, 2012); however, the precise mechanisms of these antitumor effects, particularly those on pancreatic cancer, have not been clear. In this study, we proposed a unique mechanism by which matrine has an antitumor effect by disrupting the mitochondrial metabolic function in cancer cells. We showed that matrine treatment significantly reduces ATP levels, the oxygen consumption rate, and the levels of TCA cycle intermediates and that the addition of pyruvate or α‐ketoglutarate dramatically rescues PDAC growth after suppression by matrine treatment. Thus, our data suggest that matrine can be considered to be a new therapeutic reagent targeting the various metabolic dependencies of cancer cells.

In PDAC, mutations in the oncogene KRAS are nearly universal.(Jones *et al*., 2008) In fact, KRAS mutation alone is sufficient for tumorigenesis in genetically engineered mouse models of pancreatic cancer, (Aguirre *et al*., 2003; Hingorani *et al*., 2003) and several studies have shown that KRAS must be activated for the survival of PDAC tumors.(Collins *et al*., 2012; Son *et al*., 2013; Ying *et al*., 2012) Thus, it was believed that KRAS is an ideal therapeutic target; however, the biochemical properties of this gene make it pharmacologically difficult to inhibit, and attempts to block essential post‐translational modifications of KRAS have failed clinically.(Van Cutsem *et al*., 2004) Therefore, alternative therapeutic strategies targeting the downstream pathways of the functions mediated by KRAS are critically needed. In our study, we found that matrine selectively suppresses the growth of KRAS‐mutant PDAC, but not KRAS‐wt PDAC by inhibiting autophagic flux. KRAS upregulates autophagy in PDAC to support cell proliferation and survival by maintaining energy homeostasis(Guo *et al*., 2011; Yang *et al*., 2011) and is elevated in PDAC even under nutrient‐replete conditions for macropinocytosis‐mediated glutamine supply to maintain an abundant supply of substrates for mitochondrial metabolism.(Seo *et al*., 2016) Thus, the antitumor effect of matrine on KRAS‐mutant PDAC is considered to be the mechanism by which matrine inhibits autophagic flux.

Previous studies have suggested the presence of elevated LC3‐II levels and the accumulation of autophagosomes in cancer cells in response to matrine treatment. Based on these observations, matrine has been reported to be an autophagy inducer in cancer cells, particularly pancreatic cancer cells(Liu *et al*., 2010; Zhang *et al*., 2010, 2011); however, recent studies have demonstrated that matrine disrupts autophagic flux by deacidifying lysosomes (Qiu *et al*., 2014) or by inhibiting the function of lysosomal protease.(Wang *et al*., 2013; Wu *et al*., 2017) These contrary observations could be a result of either condition‐specific effects or an incorrect assessment of autophagic flux. In fact, the increased LC3‐II levels or accumulation of autophagic vacuoles are not enough evidence to indicate autophagic flux induction because they can be a result of an increase in their formation either from this induction or from the inhibition of autophagic degradation. In this regard, we demonstrated that matrine has no significant effect on the expression levels of autophagy core genes and that increased LC3‐II levels after matrine treatment were not further increased by CQ treatment. In addition, we found that matrine inhibits lysosomal proteases by disrupting the formation of mature forms of cathepsins. Thus, our data suggest that elevated LC3‐II levels and the accumulation of autophagosomes result from the inhibition of autophagic degradation rather than from the induction of autophagic flux, which indicates that matrine inhibits autophagic flux.

Matrine exhibits pharmacological properties against various diseases, such as anti‐inflammatory, antiallergic, antivirus, antifibrotic, and cardiovascular protective effects. In addition, it exerts antitumor activities on several tumor cells by different modes of regulation. In other words, matrine influences other signaling pathways, which could account for the suppression of the HPDE growth. Our study illustrated that matrine suppresses the PDAC growth by inhibiting autophagy, thereby suggesting that autophagy inhibition could be an ideal therapeutic target for pancreatic cancer therapy.

In summary, we demonstrated that matrine suppresses PDAC growth by disrupting its mitochondrial metabolic function. Mechanistically, matrine treatment resulted in the inhibition of autophagy, which is an important mechanism by which the TCA cycle is fueled, by impairing the function of lysosomal protease. Stat3 plays an essential role in the matrine‐mediated inhibition of cathepsin maturation. In addition, we found that matrine impairs the growth of KRAS‐mutant PDAC, but not KRAS‐wt PDAC; therefore, these observations indicate that matrine can be an appropriate therapeutic reagent for KRAS‐driven cancers with high basal levels of autophagy.

## 4 Materials and methods

### 4.1 Cell culture and reagents

All human pancreatic cancer cell lines were acquired from the American Type Culture Collection and were tested regularly for mycoplasma contamination. All cells were maintained at 37 °C in humidified air with 5% CO_2_ and in Dulbecco's modified Eagle's medium (DMEM; Thermo Scientific, Waltham, MA, USA) supplemented with 10% fetal bovine serum (FBS), 100 U·mL^−1^ penicillin, and 100 μg·mL^−1^ streptomycin (Thermo Scientific, USA).

### 4.2 Cell proliferation assay

Cells were plated in 24‐well plates (density: 2000 cells/well). At the indicated time intervals, cells were fixed in 10% formalin and stained with 0.1% crystal violet. The dye was extracted with 10% acetic acid, and relative proliferation was determined according to the optical density at 595 nm.

### 4.3 Clonogenic assay

Cells were plated in 6‐well plates at 300 cells per well in 2 mL of media. The medium was not changed throughout the course of the experiment. After 10–12 days, the cell colonies were fixed in 80% methanol and stained with 0.2% crystal violet.

### 4.4 Reagents and antibodies

Matrine (M2120) was purchased from Tokyo Chemical Industry (Tokyo, Japan), and methyl pyruvate (371173), dimethyl α‐KG (349631), and CQ (C6628) were purchased from Sigma‐Aldrich (St Louis, MO, USA). Stattic (S7024) was obtained from Selleckchem (Houston, TX, USA). Antibodies to ATG3 (3415), ATG5 (2630), ATG7 (8558), P‐Stat3 (9131), and LC3 (2775) were purchased from Cell Signaling Technology (Beverly, MA, USA); antibodies to β‐actin (sc‐47778), BECN1 (sc‐11427), cathepsin D (sc‐6486), and T‐Stat3 (sc‐8019) were obtained from Santa Cruz Biotechnology (Dallas, TX, USA); antibody to cathepsin B (ab125067) was purchased from Abcam (Cambridge, MA, USA).

### 4.5 Oxygen consumption rate

The oxygen consumption rate (OCR) was measured using an XF24 extracellular flux analyzer (Seahorse Bioscience, North Billerica, MA, USA). Briefly, cells were seeded in a 24‐well Seahorse plate and cultured at 37 °C with 5% CO_2_; the medium was replaced the following day with unbuffered DMEM, and the cells were incubated at 37 °C without CO_2_ for 1 h. For measuring OCR, oligomycin, carbonyl cyanide‐4‐(trifluoromethoxy)phenylhydrazone (FCCP), and rotenone were added to reach final concentrations of 2 μm, 5 μm, and 2 μm, respectively.

### 4.6 Metabolomics

Targeted LC‐MS/MS metabolomic analysis was performed as previously described.(Seo *et al*., 2016) Briefly, the cells were grown to ~ 60% confluence in growth media on 10‐cm dishes. After 24 h of matrine treatment, the cells were washed several times with phosphate buffered saline and water, harvested using 1.4 mL cold methanol/H_2_O (80/20, v/v), and then lysed by shaking vigorously, after which 100 μL of 5‐μm internal standard was added. Metabolites were liquid–liquid extracted from the aqueous phase after adding chloroform. The aqueous phase was dried using vacuum centrifugation, and the sample was reconstituted with 50 μL 50% methanol before LC‐MS/MS analysis.

### 4.7 Quantitative reverse transcription polymerase chain reaction

Total RNA was extracted using TRIzol (QIAGEN, Hilden Germany). cDNA was synthesized from 2 μg total RNA using oligo‐dT and MMLV HP reverse transcriptase (Epicentre, Madison, WI, USA) according to the manufacturer's instruction. Quantitative reverse transcription polymerase chain reaction (qRT‐PCR) was performed on an AriaMax Real‐Time PCR instrument (Agilent Technologies, Santa Clara, CA, USA) using the SYBR detection protocol. The relative amount of cDNA was calculated using the comparative Ct method with the 18S ribosomal RNA sequences as a control. PCRs were performed in triplicate.

### 4.8 Quantitation of intracellular ATP

Intracellular ATP concentrations were measured using an ATP Colorimetric/Fluorometric Assay Kit (Biovision Incorporated, Milpitas, CA, USA) according to the manufacturer's instructions. Briefly, cells were lysed in 100 μL ATP assay buffer; 50 μL of the supernatant was collected and added to a 96‐well plate. To each well, 50 μL ATP assay buffer containing an ATP probe, ATP converter, and developer was added. Absorbance was measured at 570 nm.

### 4.9 Transmission electron microscopy

Cells were harvested by centrifugation, and the cell pellet was fixed by 2.5% glutaraldehyde and 2% paraformaldehyde in sodium cacodylate buffer (pH 7.2) at 4 °C. The fixed specimen was then postfixed in 1% osmium tetraoxide (OsO_4_) containing 1.5% potassium ferrocyanide for 30 min at 4 °C. The fixed specimen was dehydrated using an ethanol series of 50%, 60%, 70%, 80%, 90%, 100%, and 100% for 20 min in each. The specimen was subsequently transferred to Spurr's medium (Electron Microscopy Science, Hatfield, PA, USA). After impregnating the specimen with the pure resin, the tissue specimens were embedded in the same resin mixture, and samples were sectioned (60–70 nm) using an ultramicrotome (Leica UltracutUCT GmbH, Austria) and double‐stained first with 2% uranyl acetate for 20 min and then with lead citrate for 10 min. The sections were then observed under the Hitachi H7600 transmission electron microscope (Japan) at 80 kV.

### 4.10 LysoTracker staining

For LysoTracker staining, PDAC cells were plated in a 35‐mm confocal dish, which was treated with either 10 μm CQ or 2‐mm matrine the following day and then incubated for another 24 h. The cells were then stained with 50‐nm LysoTracker Red DND‐99 (Thermo Scientific, Waltham, MA, USA) for 2 h at 37 °C. The images were captured using an LSM780 confocal fluorescent microscope (ZEISS).

### 4.11 DQ‐BSA assay

Activities of lysosomal protease were measured using DQ™ Red BSA D12051 (Thermo Scientific, Waltham, MA, USA). Cells were incubated with 10 μg·mL^−1^ DQ™ Red BSA for 3 h at 37 °C prior to treatment with matrine (2 mm) and then washed with PBS for three times. The fluorescence intensity of the lysates was quantified using a VICTOR X3 2030 plate reader (Perkin Elmer, Waltham, MA, USA).

### 4.12 Xenograft studies

Female severe combined immunodeficiency mice were purchased from Charles River Laboratories. All experimental procedures were approved by the Institutional Animal Care and Use Committee of Asan Institute for Life Sciences (protocol 2017‐02‐069). Each mouse received an injection of 2 × 10^6^ cancer cells mixed with Matrigel (BD Biosciences, San Jose, CA, USA) into its flank. Five mice per group were treated when the tumor volumes reached 50–100 mm^3^. Matrine (50 mg·kg^−1^ or 100 mg·kg^−1^) was injected intratumorally into 8988T‐bearing mice once/day for 2 weeks. The length (L) and width (W) of each tumor were measured using calipers, and the tumor volume (TV) was calculated as TV = (L × W^2^)/2.

### 4.13 Statistics

Data are presented as the mean ± standard deviation. All comparisons were analyzed using the unpaired Student's *t*‐test.

## Author contributions

YC, JHL, and JS designed the study, interpreted the data, and wrote the manuscript. JHK, SL, SY, MJ, and SJK performed the experiments. HJY, CP, and JKR assisted in data interpretation.

## Supporting information


**Fig. S1.** Effect of matrine on non‐transformed human pancreatic ductal cells (HPDE) growth.
