# Supplementary material for: Matrine suppresses KRAS‐driven pancreatic cancer growth by inhibiting autophagy‐mediated energy metabolism
Source: Mol Oncol. 2018 Jun 11;12(7):1203–15. doi: 10.1002/1878-0261.12324 (PMC6026868; doi:10.1002/1878-0261.12324)
Supplement: Supplementary file 1 — Fig. S1. Effect of matrine on non‐transformed human pancreatic ductal cells (HPDE) growth. [file MOL2-12-1203-s001.pdf]

Figure S1

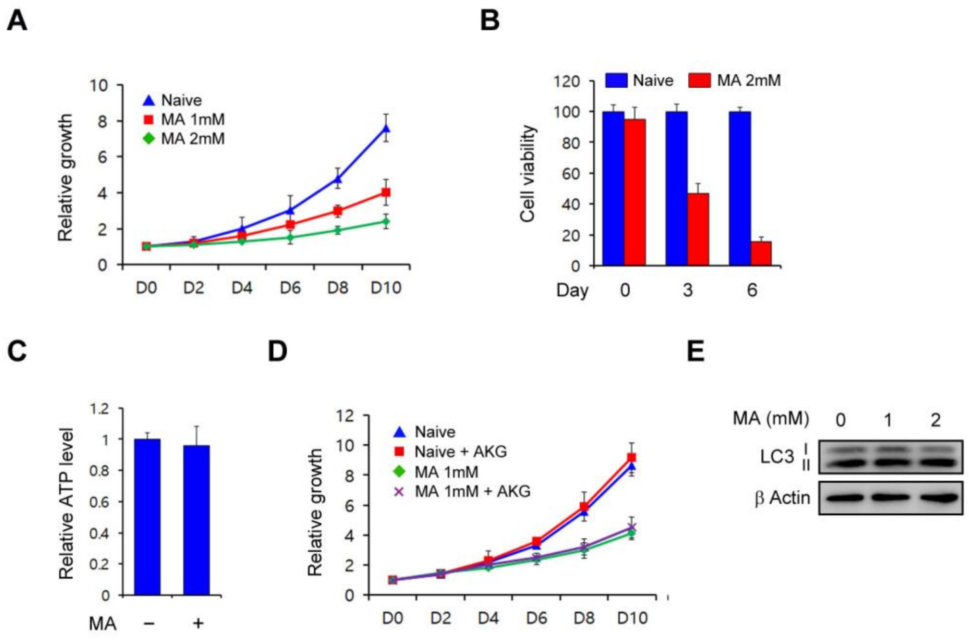

Supplementary Figure 1. Effect of matrine on non-transformed human pancreatic ductal cells (HPDE) growth. (A) HPDE cells were treated with the indicated concentration of matrine and assayed for cell growth. (B) HPDE cells were treated with 2-mM matrine for 24 h and assayed for cell viability. (C) HPDE cells were treated with 2-mM matrine for 24 h and assayed for intracellular adenosine triphosphate (ATP). (D) HPDE cells were treated with the indicated concentration of matrine in the presence of 7-mM dimethyl α-ketoglutarate and assayed for cell growth. (E) HPDE cells were treated with the indicated concentration of matrine for 24 h and immunoblotted with the indicated antibodies. Error bars represent the s.d. of triplicate wells from a representative experiment.
